# Effects of Long-Term Statin Therapy in Coronary Artery Disease Patients with or without Chronic Kidney Disease

**DOI:** 10.1155/2015/252564

**Published:** 2015-10-08

**Authors:** Huiling Huang, Chunmei Zeng, Yuedong Ma, Yili Chen, Cong Chen, Chen Liu, Yugang Dong

**Affiliations:** ^1^Department of Cardiology, First Affiliated Hospital of Sun Yat-sen University, Guangzhou 510080, China; ^2^Department of Cardiology, Yulin City First People's Hospital No. 495 Jiao Yu Zhong Road, Yuzhou, Yulin, Guangxi 537000, China; ^3^Key Laboratory on Assisted Circulation, Ministry of Health, Guangzhou 510080, China

## Abstract

*Introduction.* The effect of long-term statin therapy is essential for secondary prevention of adverse clinical outcomes of coronary artery disease (CAD) patients. No study has compared the effects of long-term statin treatment in CAD patients with or without chronic kidney disease (CKD) and CKD only patients. *Methods.* We compared the effects of long-term statin therapy (average follow-up time 5.79 years) in terms of major adverse cardiovascular events (MACE), all-cause death, and cardiac death among 570 CAD patients with or without CKD and 147 CKD only patients. *Results.* The all-cause death and cardiac death of the patients with CAD and CKD (24.4% and 20.4%) doubled those of CAD only patients (10.7% and 9.1%) (*P* < 0.001). Long-term statin therapy dramatically reduced the rates of both MACE and all-cause death/cardiac death (by 20.5% and 28.6%/27.7%, resp.) in CAD and CKD patients. CKD only patients had no significant adverse clinical outcomes and were not responsive to long-term statin therapy. *Conclusion.* Chinese CAD patients with CKD had dramatically high rates of adverse clinical outcomes; for them, long-term statin therapies were exceptionally effective in improving morbidity and mortality. CKD patients who had no cardiovascular disease initially can prognose good clinical outcomes and do not require statin treatment.

## 1. Introduction

Coronary artery disease (CAD) and its related chronic complications continue to be the leading cause of death worldwide [1]. Chronic kidney disease (CKD) has been recognized as a major and independent risk factor for cardiovascular diseases (CVD) [2–5]. Instead of progressing to end-stage renal disease (ESRD), most CKD patients die of CVD, while only 3.1% of patients progress to ESRD [6]. On the other hand, patients with CAD were frequently shown to have impaired renal function, which may contribute to worse clinical outcomes [7–10].

Statins, a family of HMG-CoA reductase inhibitors that lower cholesterol levels, are the most widely prescribed class of drugs; they are reported to reduce cardiovascular events by 25% to 45% [11]. They are well tolerated and are believed to have minimal adverse effects. These drugs are most effective for treating CVD as a secondary prevention strategy [12]. In patients with mild-to-moderate CKD, statins were found to be effective for primary prevention and to reduce cardiovascular risk. They have been reported to slow or even reverse the decline in renal function in patients with CKD, independent of lipid lowering. Multiple landmark clinical trials have demonstrated modest renoprotection with statins [13]. Thus, it is believed that statins have both cardiovascular and renal benefits in prevention and treatment.

Long-term persistence of statin therapy is essential for secondary prevention of CVD. Shalev et al. reported that better continuity of statin treatment provided an ongoing reduction in mortality among patients with and without a known history of CVD [14]. The renoprotective effect of statins on CKD patients is also shown to be dependent on the duration of treatment [14, 15]. To date, however, no study has compared the effects of long-term statin therapy in CAD patients with or without CKD after coronary artery revascularization and CKD patients, nor are there any published data available from China to address the effects of long-term statin therapy in patients with concomitant CAD and CKD. Therefore, we conducted a retrospective analysis to compare the effects of long-term statin use on cardiovascular events and mortality in three groups of Chinese patients: CAD only, CAD + CKD, and CKD only.

## 2. Methods

Hospitalization records of patients with CAD + CKD, CAD only, or CKD only were reviewed for demographic data, cardiovascular end points, and long-term regular use of statins. Inpatients were selected from the First Affiliated Hospital of Sun Yat-sen University between January 2005 and December 2008 if they were diagnosed with CAD by coronary angiogram (CAG). Patients with an estimated glomerular filtration rate (eGFR) < 60 mL/min/1.73 m^2^ were considered to have concomitant CKD (*n* = 328), while a total of 242 CAD patients with eGFR ≥ 60 mL/min/1.73 m^2^ and 147 CKD-only patients (eGFR < 60 mL/min/1.73 m^2^) were randomly selected for the control groups. The diagnosis of CAD was confirmed with coronary angiography for all patients. Patients received a stent graft or coronary artery bypass grafting surgery according to the extent of their lesions. Recorded parameters included age, gender, height, weight, history of hypertension and diabetes mellitus, and various biochemical indexes: serum creatinine, blood urea nitrogen (BUN), glycated hemoglobin (HbA1C), total cholesterol (TC), triglyceride (TG), low-density lipoprotein cholesterol (LDL-C), high-density lipoprotein cholesterol (HDL-C), apolipoprotein A-I (ApoA-I), apolipoprotein B (ApoB), apolipoprotein AI/B (ApoA-I/B), apolipoprotein E (apoE), and lipoprotein (a) (Lp-a). The results of CAG and cardiac color ultrasound and the drug therapy administered during the hospitalization were recorded and analyzed.

The participants were followed up retrospectively by medical records and telephone and were divided into the long-term statin group and statin-discontinuation (no statin) group based on the use of statins by the proportion of days covered. The primary end points (death, including cardiovascular and noncardiovascular mortality), secondary end points (revascularization, angina, nonfatal myocardial infarction, heart failure, ischemic stroke, and hemorrhagic stroke), and the most recent test results for serum creatinine, BUN, and serum lipids before the occurrence of the primary end point events were recorded. Long-term statin users were defined according to the study by Chodick et al. [16]: patients who had taken medication not less than 50% of the follow-up time minus 30 days to the end of follow-up. Otherwise, the patients were categorized into the no-statin group.

We used a simplified MDRD equation, which is more suitable for Chinese people, to evaluate kidney functions: 186 × (serum creatinine)^−1.154^  × Age^−0.203^  × (0.742 for female) [17]. The serum creatinine level was the first measurement result after the patient's admission to the hospital. The staging of renal function was categorized according to the K/DOQI guidelines: stage 3 is a moderate reduction in GFR (30–59 mL/min/1.73 m^2^), stage 4 is a severe reduction in GFR (15–29 mL/min/1.73 m^2^), and stage 5 is established kidney failure (GFR < 15 mL/min/1.73 m^2^ or ESRD period) [18]. Acute myocardial infarction, angina, and heart failure were diagnosed according to the 2008 European Society of Cardiology guidelines. Stroke was diagnosed by the 2010 Chinese guidelines for diagnosis and treatment of acute ischemic stroke [19].

Exclusion criteria included the following: (1) past diagnosis of CAD by coronary angiography in other hospitals; (2) long-term use of statins or other lipid-lowering drugs prior to admission; (3) incomplete data for lipid levels (TC, TG, HDL-C, LDL-C, ApoA-I, ApoB, and ApoA-I/B) during hospitalization; (4) patients who died during the hospitalization; (5) patients suffering from major diseases, such as severe liver disease and cancer, and expected to survive less than 3 months.

### 2.1. Statistical Analysis

The demographic and clinical characteristics were summarized by descriptive statistics. Continuous and categorical data were presented as mean ± standard deviation (SD) and number (%), respectively. For continuous variables, one-way between-groups ANOVAs were used to assess group differences. Comparisons of categorical variables were performed by the Chi-square test or the Fisher exact test. A major adverse cardiovascular event (MACE) was defined as a composite endpoint including cardiovascular events and all-cause death. The long-term event-free rate was estimated using Kaplan-Meier curves, and the log-rank test was used to identify significant differences in survival rates across the three groups. To identify the factors that might affect the incidence of MACE, a univariate Cox proportional hazard model was developed. To determine independent predictors for MACE, a multivariate Cox regression analysis was developed with the significant predictors (*P* < 0.05) and the marginally significant ones (*P* < 0.10) from the univariate model.

## 3. Results

### 3.1. Patients' Characteristics

A total of 717 patients were examined in this research. The CAD + CKD group included 328 patients. The CAD-only and CKD-only groups had 242 and 147 patients, respectively. CKD was present in 57.5% of the CAD patients.  Table 1 summarizes the characteristics of patients in the three groups. The CAD + CKD group included the oldest patients. Female gender was found more often in the CKD group. Of note, all patients in the CKD-only group had stage 5 renal disease. Hypertension and myocardial infarction were more common in the CAD + CKD group, whereas smoking and angina pectoris were found more often in the CAD group. The ejection fraction was the lowest in the CAD + CKD group. There were significant differences in concomitant medications, such as calcium-channel blockers, angiotensin-converting enzyme inhibitors (ACEI), angiotensin-receptor blockers (ARB), *β*-blockers, and aspirin across the three groups. There was also a significant difference in statin therapy: more than 90% of CAD patients with and without CKD took statins compared with only 46.3% of CKD-only patients. The three groups also differed in the baseline and follow-up laboratory results.

### 3.2. Clinical Outcomes

**Figure 1:**
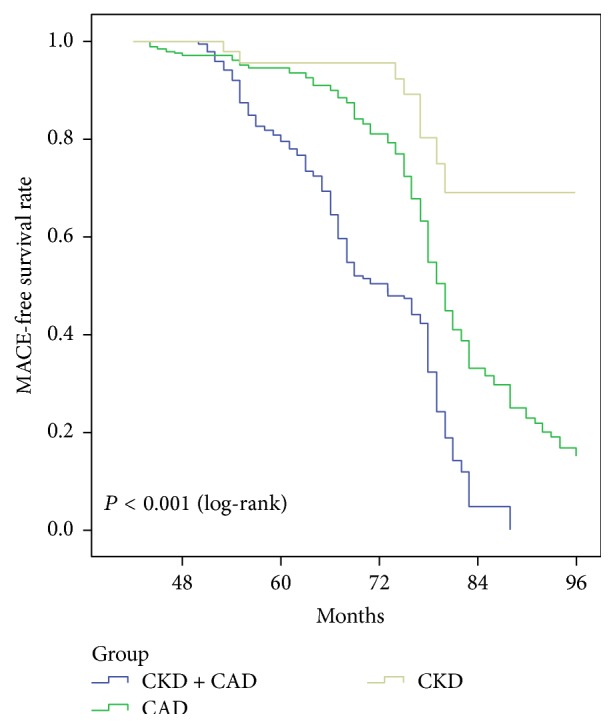
Kaplan-Meier curves for major adverse cardiovascular event- (MACE-) free survival rates for the three groups.

The CAD + CKD group was found to have cardiovascular events (55.2%), all-cause death (24.4%), and cardiac death (20.4%) with the highest frequency among the three groups (Table 2). The incidence of MACE was lowest in the CKD group (24.5%) compared with that of the CAD + CKD group (55.8%) and the CAD group (47.5%) (*P* < 0.001).  Figure 1 shows the MACE-free survival curves through 8 years for the three groups. The log-rank test revealed that the MACE-free survival rate in the CAD + CKD group was the lowest compared with that of the CAD group and the CKD group: the lowest rate occurred in the CAD + CKD group and the highest in the CKD-only group (*P* < 0.001). The median observation period for the CAD + CKD, CAD, and CKD groups was 67 months (interquartile range [IQR]: 57–77), 76 months (IQR: 69–81), and 75.5 months (IQR: 61.5–80.25), respectively.

### 3.3. Effects of Statins on Clinical Outcomes

We compared the effects of statins on the clinical outcomes of the three groups of patients (Table 3). The data revealed that long-term statin therapy was much more effective in the CAD + CKD patients compared with CAD-only and CKD-only patients. In the CAD-only patients, long-term statins exerted no significant effect on the MACE rate, while the CAD + CKD patients taking long-term statins showed a 20.5% reduction in the MACE rate (*P* < 0.001; Table 3). With regard to the all-cause and cardiac deaths, long-term statins had significant treatment effects on the CAD-only patients (reduction of about 11% in mortality rates, *P* < 0.05), but for the CAD + CKD patients, the reduction in mortality rates (about 28% reduction) was more dramatic (*P* < 0.001). In contrast, long-term statin therapy exerted no significant influence on the clinical outcomes of the CKD-only patients.

### 3.4. Factors Affecting MACE

Univariate Cox regression analysis showed that group, age, gender, hypertension, smoking, *β*-blockers, aspirin, statin discontinuation, baseline LDL-C, SBP, and DBP as well as total cholesterol, LDL-C, glucose, and SBP at final observation might affect the incidence of MACE (Table 4). Multivariate Cox regression analysis, including the significant predictors above and the marginally significant predictor of myocardial infarction in the univariate model, showed that group, smoking, statin discontinuation, baseline LDL-C, and SBP as well as total cholesterol, SBP, and glucose at final observation were independent predictors of MACE (Table 5). The CKD-only group had the lowest risk of MACE (versus CAD + CKD group: HR 0.42, 95% CI 0.31–0.57, *P* < 0.001; versus CAD-only group: HR 0.16, 95% CI 0.07–0.38, *P* < 0.001) (Table 5). The findings indicate that the CKD group had the best long-term survival status, followed by the CAD group, and the CAD + CKD group had the worst long-term survival.

## 4. Discussion

Our study is the first to compare the effects of long-term statin treatment in CAD patients with or without CKD and CKD-only patients in China. In the current study, we show that, among the Chinese CAD patients, the rate of CKD was as high as 57.5% (328/570). Reduced eGFR (<60 mL/min/1.73 m^2^) in CAD patients significantly increased the incidence of MACE and cardiovascular events. In particular, the all-cause and cardiac death rates of the CAD + CKD patients were about twice as high as the CAD-only patients. Thus, just as CKD is a risk factor for CAD, CAD is a risk factor for CKD, emphasizing that CAD and CKD are closely associated diseases with a reciprocal adverse impact. Our data show that long-term statin therapy can significantly improve the clinical outcomes of the CAD patients without CKD in terms of mortality (about a 10% reduction) but not the MACE rate. For the CAD + CKD patients, however, the treatment effect of statins was much more dramatic, with remarkable reductions in the MACE rate (20.5%), all-cause death (28.6%), and cardiac death (27.7%). In contrast, no significant treatment effect of statin was observed in the CKD-only patients. In the current study, the CAD + CKD patients had a high rate of hypertension, smoking, myocardial infarction, and angina pectoris. The unadjusted determinants of MACE included hypertension, smoking, serum LDL-C level, SBP, DBP, serum glucose level, and the use of *β*-blockers and aspirin. After adjustment, smoking, statin therapy, baseline LDL-C level, and SBP, as well as total cholesterol levels, SBP, and glucose at final observation were significant predictors of MACE. These findings are consistent with previous reports in general [7, 10, 20].

Few studies have examined the effects of long-term statins on clinical outcomes of CAD patients with or without renal insufficiency after coronary revascularization, and no such data from China were previously available. Moreover, to our knowledge, this is the first study in any location to have compared the effects of long-term statin therapy in the three groups of patients (CAD with or without CKD and CKD only). Kaneko et al. [20] studied the effects of long-term statins on CAD patients with renal insufficiency but did not include the CAD-only and CKD-only patients. Another Japanese group [21] compared the effects of statins on the clinical outcomes of CAD patients with or without renal insufficiency; however, there was no CKD-only patient group in their investigation, and the duration of statin treatment was shorter (3 years). Longer-term statin therapy may exert more significant treatment effects, and our results show that the extent of reduction in MACE, all-cause deaths, and cardiac deaths appears to be more dramatic compared with the Japanese studies. Noticeably, all CAD patients in our study, with or without CKD, had remarkably higher rates of MACE and fatal events compared with those in Japan; these higher rates would be subject to more significant treatment effects of statins. This suggests that the Chinese CAD patients are substantially different from those in Japan. Possible reasons for the high morbidity and mortality in China include worse economic conditions, heavier labor, and insufficient medical care. Thus, in CAD patients with worse clinical outcomes like in China, long-term statin therapy can exert more dramatic treatment effects, and measures should be taken to enhance adherence to statins.

A surprising finding is that the CKD patients without CAD had very low all-cause or cardiac mortality, and statin therapy did not have a significant impact on the cardiovascular events and mortality rates. Statin treatment has been shown to be beneficial for CKD patients not undergoing dialysis [13, 15, 22, 23]; however, previous studies have not separated CKD patients with or without CVD. In the current study, we selectively analyzed the CKD patients who did not have CVD at registration; these patients did not develop significantly increased cardiovascular events and mortality rates during the long follow-up period nor did they show an effective response to statin treatment. Thus, CKD patients free of CVD at baseline appear to have an excellent prognosis and, therefore, may not need statin treatment. This finding may provide a significant guideline regarding statins in CKD patients, and it warrants larger scale studies across multiple clinical centers in China.

We also found that CKD patients had a high rate of statin discontinuation. According to Morrison and colleagues, the most common reason for long-term discontinuation of lipid-lowering medications in CKD patients is adverse reactions [24]. However, due to the retrospective nature of our study design, it was not possible to explore the potential reasons behind statin discontinuation in the current research. Of note is that there is evidence to suggest that a high incidence of adverse events (e.g., myopathy) is associated with statins in ESRD patients [25] though there is also a lack of agreement regarding the administration/discontinuation of statins in patients with ESRD [26].

In patients with stage 5 CKD, Pennell and colleagues found that the prevalence of dyslipidemia was 82%, which was characterized by raised triglycerides and very-low-density lipoproteins and decreased levels of high-density lipoproteins [27]. Due to increased oxidative stress, furthermore, CKD is associated with accelerated progression of atherosclerosis [28]. Thus, there are reasons to consider statin treatment in CKD patients. There are a number of studies exploring the effect of statins in different CKD stages, but the findings are inconclusive. On one hand, there is evidence in support of the effect of statins in reducing cardiovascular events in CKD patients, irrespective of the stage [23, 29]. On the other hand, Palmer and colleagues indicated that statins had little effect in patients undergoing dialysis [13]. The majority of patients with CAD + CKD in the current study had end-stage renal disease with an enhanced predisposition to accelerated atherosclerosis and, thus, would derive the greatest benefit from the statin therapy.

Limitations of the current study include a lack of records on statin types and dosage that precluded an analysis of the effect of these factors on clinical outcomes and lack of information on patients with CKD stages 1 to 4 that precluded an analysis on the effect of statins in different CKD stages. Therefore, a more carefully controlled prospective study would be necessary to achieve this purpose. Another limitation is that it is a clinical study from a single center. Future studies that include a larger cohort of patients from multiple centers in China are necessary to corroborate our findings and to support their generalizability.

## 5. Conclusions

This study is the first to compare clinical outcomes in Chinese CAD patients with or without CKD and in CKD-only patients treated with long-term statins; it provides new insights about the appropriate treatment of these three groups of patients. More than half of the CAD patients in this study had chronic kidney disease, which greatly contributed to the adverse clinical outcomes of the CAD patients. Long-term statin therapy significantly improved the mortality rate but not the MACE rate of the CAD patients without renal insufficiency, while it more dramatically reduced the MACE and mortality rates of the CAD patients with concomitant CKD. In contrast, CKD-only patients had a good prognosis and did not appear to require statin treatment.

## Figures and Tables

**Table 1:** Patients' characteristics.

Variables	CAD + CKD	CAD	CKD	*P* value
	(*n* = 328)	(*n* = 242)	(*n* = 147)	
*Characteristics*				
Age (years)	69.7 ± 8.4	61.6 ± 10.5	53.6 ± 17.8	<0.001
BMI (kg/m^2^)	24.2 ± 2.2	23.2 ± 2.2	23.3 ± 2.3	<0.001
Male	232/328 (70.7)	171/242 (70.7)	64/147 (43.5)	<0.001
Hypertension	246/328 (75.0)	132/242 (54.5)	83/147 (56.5)	<0.001
Diabetes mellitus	88/328 (26.8)	71/242 (29.3)	42/147 (28.6)	0.843
Smoking	150/328 (45.7)	123/242 (50.8)	25/147 (17.0)	<0.001
Myocardial infarction	186/328 (56.7)	114/242 (47.1)	0/147 (0)	<0.001
Angina pectoris	133/328 (40.5)	123/242 (50.8)	0/147 (0)	<0.001
Stroke	20/328 (6.1)	14/242 (5.8)	8/147 (5.4)	0.992
Ejection fraction	61.8 ± 14.2	65.6 ± 8.8	63.2 ± 12.7	0.017
Statin therapy	301/328 (91.8)	226/242 (93.4)	68/147 (46.3)	<0.001
*Concomitant medications*				
Calcium-channel blocker	115/328 (35.1)	58/225 (25.8)	75/147 (51.0)	0.004
ACEI	148/328 (45.1)	103/242 (42.6)	28/147 (19.0)	0.002
ARB	119/328 (36.3)	83/242 (34.3)	77/147 (52.4)	0.041
*β*-blocker	236/328 (72.0)	190/242 (78.5)	44/147 (29.9)	<0.001
Loop diuretic	50/328 (15.3)	13/121 (10.7)	14/147 (9.5)	0.36
Aspirin	302/328 (92.1)	228/242 (94.2)	44/147 (29.9)	<0.001
*Clinical parameters*				
Total cholesterol (mmol/l)	4.7 ± 1.2	4.8 ± 1.1	6.5 ± 2.9	<0.001
LDL-C (mmol/l)	9.7 ± 119.1	4.2 ± 17.9	3.9 ± 2.4	0.746
HDL-C (mmol/l)	1.0 ± 0.3	1.0 ± 0.3	1.2 ± 0.4	<0.001
Triglycerides (mmol/l)	1.6 ± 1.0	1.7 ± 1.1	2.4 ± 1.5	<0.001
HbA1c (%)	6.4 ± 1.2	6.6 ± 1.4	7.8 ± 2.1	<0.001
Glucose (mmol/l)	7.2 ± 4.5	6.5 ± 4.1	14.0 ± 55.3	0.014
SBP (mmHg)	136.8 ± 30.0	130.1 ± 17.7	138.3 ± 22.4	0.006
DBP (mmHg)	78.0 ± 15.9	78.9 ± 11.2	73.5 ± 10.0	0.041
BUN (mmol/l)	8.8 ± 4.3	5.6 ± 1.5	12.3 ± 6.5	<0.001
Serum creatinine (*μ*mol/l)	155.0 ± 111.8	80.5 ± 15.3	275.4 ± 205.9	<0.001
ApoA-I (g/l)	1.3 ± 3.9	1.0 ± 0.3	1.3 ± 0.4	0.588
ApoB (g/l)	1.0 ± 2.3	0.9 ± 0.2	1.0 ± 0.4	0.638
ApoA-I/B	1.3 ± 0.5	1.5 ± 2.8	1.4 ± 0.7	0.381
ApoE (ng/dl)	43.6 ± 18.7	43.9 ± 26.0	97.7 ± 590.3	0.119
Lp (a) (ng/ml)	383.1 ± 327.2	376.2 ± 429.3	372.3 ± 395.6	0.955
*Clinical parameters at final observation*				
Total cholesterol (mmol/l)	4.8 ± 1.3	4.4 ± 1.1	5.0 ± 1.2	<0.001
LDL-C (mmol/l)	2.9 ± 1.1	2.7 ± 0.9	3.1 ± 1.0	0.005
HDL-C (mmol/l)	1.1 ± 0.3	1.1 ± 0.3	1.1 ± 0.2	0.551
Triglycerides (mmol/l)	1.6 ± 1.0	1.6 ± 1.0	1.7 ± 1.0	0.69
HbA1c (%)	6.6 ± 1.2	6.8 ± 1.3	5.9 ± 0.7	0.029
Glucose (mmol/l)	7.1 ± 3.0	6.4 ± 2.1	6.5 ± 2.3	0.011
SBP (mmHg)	138.2 ± 27.7	128.7 ± 16.1	134.7 ± 22.1	<0.001
DBP (mmHg)	78.1 ± 13.9	78.2 ± 10.9	82.4 ± 10.7	0.074
BUN (mmol/l)	10.6 ± 7.2	6.0 ± 1.7	13.9 ± 6.8	<0.001
Serum creatinine (*μ*mol/l)	225.1 ± 168.8	83.6 ± 20.9	349.1 ± 255.0	<0.001
ApoA-I (g/l)	1.6 ± 6.7	1.2 ± 0.5	1.1 ± 0.3	0.570
ApoB (g/l)	0.9 ± 0.3	0.8 ± 0.2	1.0 ± 0.4	<0.001
ApoA-I/B	1.4 ± 0.6	1.6 ± 0.7	1.3 ± 0.6	<0.001
ApoE (ng/dl)	43.4 ± 28.4	40.9 ± 16.6	53.3 ± 66.6	0.010
Lp (a) (ng/ml)	444.5 ± 1003.7	404.4 ± 401.3	346.9 ± 261.2	0.452

Values are expressed as number of patients (percentage) or mean ± SD.

ACEI, angiotensin-converting enzyme inhibitor; ARB, angiotensin-receptor blocker; ApoA-I, apolipoprotein A-I; ApoB, apolipoprotein B; ApoE, apolipoprotein E; BMI, body mass index; BUN, blood urea nitrogen; CAD, coronary artery disease; CKD, chronic kidney disease; DBP, diastolic blood pressure; HbA1c, hemoglobin A1c; HDL-C, high-density lipoprotein cholesterol; LDL-C, low-density lipoprotein cholesterol; Lp (a), lipoprotein (a); SBP, systolic blood pressure.

**Table 2:** Clinical outcomes.

Variables	CAD + CKD	CAD	CKD	*P* value
	(*n* = 328)	(*n* = 242)	(*n* = 147)	
*Clinical outcomes*				
MACE	183 (55.8)	115 (47.5)	36 (24.5)	<0.001
Cardiovascular events	181 (55.2)	110 (45.5)	36 (24.5)	<0.001
All-cause death	80 (24.4)	26 (10.7)	9 (6.1)	<0.001
Cardiac death	67 (20.4)	22 (9.1)	4 (2.7)	<0.001

Values are expressed as number of patients (percentage).

CAD, coronary artery disease; CKD, chronic kidney disease; MACE, major adverse cardiovascular event.

**Table 3:** Effects of statins on clinical outcomes of different group of patients.

Variables	Long-term statins	No statin	Extent of reduction	*P* value
*CAD-only patients*				
MACE	89/197 (45.2%)	21/45 (46.7%)	1.5%	0.987
All-cause death	17/197 (8.6%)	9/45 (20%)	11.4%	0.049
Cardiac death	14/197 (7.1%)	8/45 (17.8%)	10.7%	0.049
*CAD + CKD patients*				
MACE	77/170 (45.3%)	104/158 (65.8%)	20.5%	<0.001
All-cause death	18/170 (10.6%)	62/158 (39.2%)	28.6%	<0.001
Cardiac death	12/170 (7.1%)	55/158 (34.8%)	27.7%	<0.001
*CKD-only patients *				
MACE	14/41 (34.1%)	22/106 (20.8%)	−13.3%	0.142
All-cause death	2/41 (4.9%)	7/106 (6.6%)	1.7%	0.99
Cardiac death	0/41 (0%)	4/106 (3.8%)	3.8%	0.48

CAD, coronary artery disease; CKD, chronic kidney disease; MACE, major adverse cardiovascular event.

**Table 4:** Unadjusted predictors for MACE.

	Hazard ratio	95% CI	*P* value
CKD versus CAD + CKD	0.43	(0.34, 0.55)	<0.001
CKD versus CAD	0.14	(0.07, 0.28)	<0.001
Age (years)	1.03	(1.01, 1.04)	<0.001
Male (versus female)	0.78	(0.61, 0.99)	0.045
Hypertension	1.42	(1.11, 1.82)	<0.01
Diabetes mellitus	1.21	(0.94, 1.54)	0.134
Smoking	1.42	(1.13, 1.79)	<0.01
Myocardial infarction	1.25	(1.0, 1.57)	0.055
Angina pectoris	1.13	(0.90, 1.42)	0.28
Stroke	1.44	(0.91, 2.27)	0.118
Ejection fraction	0.99	(0.98, 1.00)	0.165
Calcium-channel blocker	1.13	(0.88, 1.44)	0.346
ACEI	1	(0.80, 1.26)	0.983
ARB	0.94	(0.75, 1.19)	0.627
*β*-blocker	1.49	(1.14, 1.94)	<0.01
Loop diuretic	1.15	(0.79, 1.66)	0.475
Aspirin	2.21	(1.40, 3.48)	<0.01
*Clinical parameters at baseline*			
Total cholesterol (mmol/l)	0.96	(0.87, 1.05)	0.319
LDL-C (mmol/l)	1.002	(1.001, 1.003)	<0.001
HDL-C (mmol/l)	0.78	(0.52, 1.19)	0.251
Triglycerides (mmol/l)	0.98	(0.88, 1.08)	0.65
HbA1c (%)	0.98	(0.88, 1.09)	0.728
Glucose (mmol/l)	1	(0.99, 1.01)	0.614
SBP (mmHg)	0.994	(0.99, 1.0)	0.011
DBP (mmHg)	0.99	(0.98, 1.0)	0.011
*Clinical parameters at final observation*			
Total cholesterol (mmol/l)	1.31	(1.19, 1.44)	<0.001
LDL-C (mmol/l)	1.26	(1.13, 1.41)	<0.001
HDL-C (mmol/l)	1.04	(0.68, 1.60)	0.846
Triglycerides (mmol/l)	1.09	(0.97, 1.21)	0.135
HbA1c (%)	1.02	(0.92, 1.14)	0.702
Glucose (mmol/l)	1.1	(1.06, 1.15)	<0.001
SBP (mmHg)	1.006	(1.0, 1.01)	0.046
DBP (mmHg)	0.997	(0.99, 1.01)	0.545
Statins	0.247	(0.19, 0.38)	<0.001

ACEI, angiotensin-converting enzyme inhibitor; ARB, angiotensin-receptor blocker; CAD, coronary artery disease; CKD, chronic kidney disease; DBP, diastolic blood pressure; HbA1c, hemoglobin A1c; HDL-C, high-density lipoprotein cholesterol; LDL-C, low-density lipoprotein cholesterol; MACE, major adverse cardiovascular event; SBP, systolic blood pressure.

**Table 5:** Adjusted determinants of MACE.

	Hazard ratio	95% CI	*P* value
*Group*			
CKD versus CAD + CKD	0.42	(0.31, 0.57)	<0.001
CKD versus CAD	0.16	(0.07, 0.38)	<0.001
Age (years)	1.01	(1.0, 1.03)	0.065
Male (versus female)	1.23	(0.91, 1.68)	0.183
Hypertension	1.29	(0.99, 1.68)	0.061
Smoking	1.58	(1.19, 2.08)	0.002
Myocardial infarction	1.04	(0.80, 1.34)	0.791
*β*-blocker	1.13	(0.83, 1.53)	0.449
Aspirin	1.19	(0.72, 1.98)	0.505
*Clinical parameters at baseline*			
LDL-C (mmol/l)	1.001	(1.0, 1.002)	0.008
SBP (mmHg)	0.99	(0.98, 0.994)	<0.001
DBP (mmHg)	1	(0.99, 1.01)	0.88
*Clinical parameters at final observation*			
Total cholesterol (mmol/l)	1.38	(1.13, 1.68)	0.002
LDL-C (mmol/l)	0.89	(0.70, 1.13)	0.334
SBP (mmHg)	1.01	(1.001, 1.013)	0.023
Glucose (mmol/l)	1.06	(1.01, 1.12)	0.03
Statins	0.25	(0.2, 0.38)	<0.001

CAD, coronary artery disease; CKD, chronic kidney disease; DBP, diastolic blood pressure; LDL-C, low-density lipoprotein cholesterol; MACE, major adverse cardiovascular event; SBP, systolic blood pressure.
